# Kinesiophobia Predicts Physical Function and Physical Activity Levels in Chronic Pain-Free Older Adults

**DOI:** 10.3389/fpain.2022.874205

**Published:** 2022-04-27

**Authors:** Kelly M. Naugle, Corinthian Blythe, Keith E. Naugle, NiCole Keith, Zachary A. Riley

**Affiliations:** Department of Kinesiology, School of Health and Human Sciences, Indiana University Purdue University Indianapolis (IUPUI), Indianapolis, IN, United States

**Keywords:** kinesiophobia, physical function, physical activity, older adults, activity-related pain

## Abstract

Advanced aging is associated with a general decline in physical function and physical activity. The current evidence suggests that pain-related fear of movement (i.e., kinesiophobia) is increased in the general older adult population and impacts physical activity levels in patients with chronic pain. However, whether kinesiophobia could impact physical activity and function in relatively healthy, chronic pain-free older adults remain unclear. Thus, the purpose of this study was to examine whether fear of movement due to pain predicted self-reported and objective levels of physical function and physical activity in healthy older adults without chronic pain. Fifty-two older adults were enrolled in this study. The participants completed the International Physical Activity Questionnaire (IPAQ) and wore an accelerometer on the hip for 7 days to measure physical activity. Measures of sedentary time, light physical activity, and moderate to vigorous physical activity were obtained from the accelerometer. Measures of physical function included the Physical Functioning subscale of the Short Form-36, Short Physical Performance Battery (SPPB), the 30-s Chair Stand test, and a maximal isometric hand-grip. The Tampa Scale of Kinesiophobia (TSK) was used to measure fear of movement or re-injury associated with pain. Potential covariates included self-reported activity-related pain and demographics. Hierarchical linear regressions were conducted to determine the relationship of kinesiophobia with levels of physical activity and physical function while controlling for activity-related pain and demographics. TSK scores did not predict self-reported physical activity on the IPAQ. However, TSK scores predicted self-reported physical function (Beta = −0.291, *p* = 0.015), 30-s Chair Stand test scores (Beta = −0.447, *p* = 0.001), measures from the SPPB (Gait speed time: Beta = 0.486, *p* < 0.001; Chair stand time: Beta = 0.423, *p* = 0.003), percentage of time spent in sedentary time (Beta = 0.420, *p* = 0.002) and light physical activity (Beta = −0.350, *p* = 0.008), and moderate to vigorous physical activity (Beta = −0.271, *p* = 0.044), even after controlling for significant covariates. These results suggest that greater pain-related fear of movement/re-injury is associated with lower levels of light and moderate to vigorous physical activity, greater sedentary behavior, and worse physical function in healthy, chronic pain-free older adults. These findings elucidate the potential negative impact of kinesiophobia in older adults who don't report chronic pain.

## Introduction

Substantial research has identified physical inactivity in older adults as a modifiable risk factor for all-cause mortality (1), dementia (2), type 2 diabetes (3), depression (4), and reduced health-related quality of life (5, 6). Despite the well-known risks of physical inactivity for older adults, physical activity declines across the age span with almost one-third of adults aged 50 or older reported as inactive (7, 8). Thus, it is imperative to understand factors that influence physical activity behavior in older adults.

Research has identified several barriers to physical activity participation in older adults including (but not limited to) lack of motivation (9, 10), fear of falling, environmental barriers (10), and physical limitations due to existing medical conditions and/or pain (9, 11, 12). Indeed, several lines of research suggest that pain adversely affects the physical activity behavior of older adults, often leading to performance difficulties or termination of higher-order physical activities (e.g., household maintenance, climbing stairs, yard work, and walking long distances) (12). Importantly, pain is not only a physiological/sensory sensation but also includes emotional and cognitive components that can combine to facilitate a fear of pain (13). Prior studies in chronic pain populations have revealed that pain-related fear of movement is associated with reduced physical activity and physical function, over and beyond the effect of actual pain (14, 15). The phenomenon of fear of movement or (re)injury related to pain is referred to as kinesiophobia (16) and has most commonly been measured with the Tampa Scale of Kinesiophobia (TSK) (17).

Kinesiophobia could be a factor contributing to the limited and variable physical activity participation in older adults. Notably, a large study of the general Finnish population revealed higher kinesiophobia in men over 55 years and women over 65 years compared with younger adults (18). In the entire sample, those reporting lower leisure-time physical activity had higher kinesiophobia scores. However, leisure-time physical activity was measured with a single question, and relationships were not examined within different age groups. Importantly, no studies have examined the relationships between physical activity, physical function, and kinesiophobia specifically in non-clinical older adult populations. Therefore, although kinesiophobia appears to be increased in the general older adult population and impacts chronic pain patients, it remains unclear whether kinesiophobia could impact physical activity and function in relatively healthy, chronic pain-free older adults.

Another limitation within the kinesiophobia literature is that many studies have relied on self-reported physical activity and disability, which represent a participant's perception of his/her own function and activity. Self-reported physical activity is vulnerable to under or over-reporting, human error, and recall. Additionally, physical function is multidimensional and the Initiative on Methods, Measurement, and Pain Assessment in Clinical Trials (IMMPACT) advises that physical function assessment include patient-reported outcomes, performance-based measures (i.e., functional capacity during a standardized test), and objective measures of physical activity (i.e., ambulatory measurements with accelerometers) (19). Very little research focused on kinesiophobia has included these different types of physical function measurement within one study.

In summary, advanced aging is associated with a general decline in physical function and physical activity (7), but how kinesiophobia is related to this natural decline is still unknown. Thus, the purpose of this study was to examine whether kinesiophobia predicted self-reported levels of physical function and activity, performance-based physical function, and objective levels of physical activity *via* accelerometry in healthy, chronic pain-free older adults. We hypothesized that older adults reporting relatively greater pain-related fear of movement (i.e., kinesiophobia) would exhibit decreased physical function, greater sedentary behavior, and less physical activity.

## Methods

### Participants

The participants were 52 healthy adults ranging in age from 60 to 77 (males = 20, females = 32). Table 1 presents the descriptive characteristics of the sample. The racial composition of the sample included 47 Caucasians, 1 Hispanic, and 4 African Americans. The participants were recruited through posted advertisements in the local community. Individuals meeting any of the following criteria, based on self-report, were excluded from the study: (1) current use of narcotics or any tobacco products, chronic use of analgesics, (2) serious systemic disease or condition that restricted normal daily activities (e.g., cancer, severe osteoarthritis), (3) cognitive impairment that would interfere with understanding of the study procedures as defined by a score of >7 on the Six Item Cognitive Impairment Test, (4) uncontrolled hypertension, (5) cardiovascular, metabolic, or pulmonary disease, (6) neurological disease, (7) serious psychiatric conditions (e.g., schizophrenia and bipolar disorder), and (8) chronic pain or any ongoing pain problem (headaches, injury-related pain, etc.).

**Table 1:** Descriptive statistics for primary study measures in male and female participants.

**Variable**	**Women**	**Men**	***p*** **-value**
	**(*n* = 31)**	**(*n* = 20)**	
Age, year	67.0 (76, 58)	66.5 (75.5, 57.5)	0.797
BMI	24.3 (29.1, 19.5)	26.6 (31.2, 22.1)	0.193
% of sedentary time per day, %	65.5 ± 6.7	70.0 ± 7.6	0.058
% of LPA per day, %	32.1 ± 6.2	27.2 ± 6.9	0.019^*^
MVPA per day, minutes	20.5 (42.7, −1.7)	23.5 (64.1, −17.1)	0.275
Handgrip MVC	49.8 (64.3, 35.2)	81.4 (128, 34.8)	<0.001^*^
SPPB–Gait subscore (0–4)	4.0 (4, 4)	4.0 (4, 4)	0.292
SPPB–Gait speed, seconds	3.3 (4, 2.6)	3.3 (4, 2.6)	0.425
SPPB–Chair stand subscore (0–4)	4 (5, 3)	4 (5.7, 2.2)	0.309
SPPB–Chair stand time, seconds	10.7 (14.5, 6.9)	10.3 (15.2, 5.4)	0.565
SPPB–Balance score (0–4)	4.0 (4, 4)	4.0 (4, 4)	0.561
SPPB–Total Score (0–12)	12 (13, 11)	12 (15, 9)	0.486
30-s Chair stand test score, reps	13.5 (18.2, 8.7)	14.5 (22.5, 6.5)	0.963
Tampa scale of kinesiophobia (11–44)	17.5 (23.2, 11.7)	18 (23, 13)	0.323
Activity related pain index, z-score	0.86 (3.74, −2.02)	0.80 (4.16, −2.56)	0.712
SF-36 Physical function subscale, (0–100)	90 (100, 80)	90 (105, 75)	0.940
IPAQ–Vig activity, METs-minute/week	480 (2,400, −1,440)	1,200 (3,600, −1,200)	0.080
IPAQ–Mod activity, METs-minute/week	2,655 (6,399, −1,089)	1,575 (5,715, −2,565)	0.894
IPAQ–Walking, METS-minute/week	907.5 (2,986.5, −1,171.5)	1,386 (4,554, −1,782)	0.304
IPAQ–Sit subscale, minutes/week	540 (892.5, 187.5)	600 (1080, 120)	0.231
IPAQ–Total, METS-minute/week	5,089 (10,208.5, −30.5)	6,474 (16,926, −3,978)	0.235

* *= significant sex difference at the p = 0.05 level. Descriptives are presented as medians and percent quartiles for non-normally distributed variables, and means and standard deviations for normally distributed variables. BMI, Body mass index; LPA, Light physical activity; MVPA, moderate to vigorous physical activity; IPAQ, International Physical Activity Questionnaire; SPPB, Short Physical Performance Battery; MVC, Maximal voluntary contraction*.

### Procedures

This study was approved by the Indiana University Human Subject Review Board. Participants completed a screening/orientation session and three experimental sessions. All sessions were scheduled on separate days and separated by at least 48 h. Additionally, participants were instructed to refrain from consuming caffeinated beverages or any pain medications (e.g., Tylenol) prior to the experimental sessions. During the experimental sessions, participants completed several questionnaires, completed tests of physical functioning, and underwent quantitative sensory testing (QST: the results of the QST are reported elsewhere and not the focus of this study). The handgrip test was performed during the orientation session, while the Short Physical Performance Battery (SPPB) and the 30-s chair stand test were performed during separate experimental sessions. Additionally, physical activity behavior for 1 week was assessed with an accelerometer. These assessments are described below.

### Screening and Orientation Session

The screening and orientation session lasted approximately 2 h and occurred on a separate day from the experimental sessions. All participants were provided information about the experimental procedures, and reviewed and signed an informed consent form prior to participation in the study. To determine eligibility, participants completed a health history questionnaire, supplemented by an interview, blood pressure, and height and weight measurements. The participants were also administered the Six-item Cognitive Impairment Test to ensure that participants were free of cognitive impairment that would compromise study participation (20). No participants were excluded following the orientation and training session. Participants also completed an assessment of maximal voluntary contraction (MVC) of handgrip muscles. At the end of the training session, participants were given an accelerometer, instructions on how to wear the device, and a physical activity diary (described further below).

### Objective Measures of Physical Activity

All participants were instructed to wear an accelerometer (Actigraph GT3X+) on the hip to measure physical activity levels. The Actigraph is a small lightweight tri-axial accelerometer that is designed to detect tri-axial accelerations in the range of 0.05-2 G. Output from the ActiGraph is in the form of step counts, body positions, and activity counts for a specific time period. Data were captured in 1-min epochs, and non-wear time was defined as 60 min of consecutive zero counts (21). Participants were given the accelerometer and instructions on how to wear it during the screening session. They were instructed to wear the accelerometer for 7 consecutive days following the screening session except during sleep, showering/bathing, and swimming. A valid collection period per participant was defined as having worn the device for more than 10 h per day for ≥ 4 days (21). Participants were also provided a Physical Activity Diary in which they recorded the start and finish times each day, as well as the duration and reason for any periods where they took the accelerometer off. Participants received reminder calls or emails from research staff about wearing the accelerometers.

Data obtained from the accelerometer were processed by the ActiLife Data Analysis Software (Actigraph, Pensacola, FL, United States). Activity count cut-points to determine the amount of time a participant spent in sedentary, light, or moderate to vigorous activity were defined as <100 counts/min (sedentary), 100–1,951 counts/min (Light physical activity), and >1,951 counts/min (moderate to vigorous activity), respectively (22). These cut-points have been used by other studies to measure physical activity behavior in older adults (23–25). Moderate physical activity and vigorous physical activity were combined into MVPA because very few older adults actually performed vigorous physical activity according to the accelerometer data. MVPA was expressed in min/day. Light physical activity and sedentary time are highly related to accelerometer wear time. Therefore, the measure of light physical activity used for data analysis was defined as the percentage of accelerometer wear time that the participant spent in light physical activity. Similarly, the measure of sedentary time used for the data analyses was defined as the percentage of accelerometer wear time that the participant spent in sedentary time.

### Performance-Based Measures of Physical Function

#### Maximal Voluntary Contraction on Hand Grip

The MVC of the right-hand flexor muscles was determined with a hand dynamometer (Jamar Plus digital hand dynamometer; Patterson Medical, China). The dynamometer handle was adjusted according to the manufacturer's guidelines for each participant. The participants placed their right arm on a stable surface with the elbow at a 90°angle and firmly gripped the hand dynamometer. The participants were asked to squeeze the dynamometer as hard as possible for 5 s. This procedure was repeated three times with a 1-minute rest between trials. The high score of the three MVCs was used as the MVC. Handgrip strength using the Jamar device has been shown to have good to excellent reliability in older adults (26, 27).

#### SPPB

Lower-extremity physical function was assessed using the SPPB (28). The SPPB has been used over the past 2 decades to objectively characterize physical function in older persons. The SPPB consists of a 4-meter walk, repeated chair stands, and tests of standing balance. The performance on each of the three tasks was assigned a categorical score, ranging from 0 to 4. The categorical scores were summed for a Total SPPB score. The reliability of the SPPB is high (ICC's 0.88–0.92) and its validity for measuring lower-extremity physical function has been well-established (29–31). Below is a description of the 3 SPPB tasks.

##### 4-Meter Walk

The participants walked a straight 4-meter course at their usual pace. The 4-meter walk was timed with a stopwatch and completed two times. The average time in seconds of the two trials was used for data analysis.

##### Repeated Chair Stands

Subjects were asked to fold their arms across their chest and to stand up once from a chair. If successful, they were asked to stand up and sit down five times as quickly as possible. The repeated chair stands were timed with a stopwatch. The procedure stopped if the participant used arms, or after 1 min if the participant had not completed the rises. The completion time in seconds of the 5 chair stands was used for data analysis.

##### Balance Testing

Subjects were asked to maintain the side-by-side (stand with feet together side by side), semi-tandem (stand with the side of the heel of one foot touching the big toe of the other foot), and tandem (stand with the heel of one foot in front of and touching the toes of the other foot) positions for 10 s. Participants were assigned a score based on performance.

#### 30-s Chair Stand Test

The 30-s Chair Stand Test is one aspect of the Senior Fitness Test (32) that the American College of Sports Medicine recommends can be used to safely and effectively assess muscular strength and endurance in most older adults (33). While this test is similar to the chair stand test used in the SPPB, research in older adults suggests that the physical construct represented by these two chair stand tests may not be identical. For example, the 30-s chair stand test better reflects muscular endurance, while the SPPB chair stand test is more dependent on dynamic balance (34). Thus, we decided to include both chair stand tests. The 30-s Chair Stand test was administered using a folding chair without arms, placed against a wall to prevent it from moving during the test. The test begins with the participant seated in the middle of the chair, feet at an angle slightly back from the knees, with one foot slightly in front of the other to help maintain balance when standing. Arms were crossed at the wrists and held against the chest. At the signal of “go,” the participant rose to a full stand and then returned back to the initial position. The participants were encouraged to complete as many full stands as possible within a 30-s time limit. The score for data analysis is the number of correctly executed stands within the 30-s limit.

### Self-Report Questionnaires

#### Kinesiophobia

Tampa Scale of Kinesiophobia-11 (TSK) consists of 11 items used to measure fear of movement or re-injury associated with pain (35, 36). Examples of items on the TSK include “Pain always means I have injured my body” and “Pain lets me know when to stop exercising so that I don't injure myself.” These items are rated on a 4-point Likert scale ranging from *strongly disagree* to *strongly agree*. The TSK is a reliable and valid method for determining fear of movement in both clinical and non-clinical populations (37, 38). The total TSK score ranges from 11 to 44, with higher scores indicating greater fear of movement due to pain.

#### Activity-Related Pain

Even though the study sample reported no chronic pain, older adults may still experience some pain, especially during movement. Therefore, an activity-related pain measure was included so our analyses could evaluate the effects of fear of movement, after controlling for actual pain with movement. Participants completed the pain scale of the Quality of Wellbeing Scale- Self-administered (QWB-SA) (39, 40). The QWB-SA is a generic measure of health-related quality of life (HRQOL) that combines preference-weighted values for symptoms and functioning. The measure has been used in multisite NIH clinical trials and with various medical conditions (41, 42). The QWBA-SA includes a 12-item pain scale. The first six questions ask participants to indicate “*how often he/she experienced pain in the past week while doing the following activities*”: (1) getting in and out of bed, (2) walking a short (1 block) distance, (3) getting in and out of a chair, (4) walking up a flight of stairs, (5) getting in and out of a car, and (6) walking down a flight of stairs. Participants rate the frequency of pain on a 5-point Likert scale ranging from *always* to *never*. The remaining 6 questions ask participants to indicate the severity of pain experienced while doing the same activities. Pain severity was rated on a 6-point Likert scale ranging from *no pain* to *excruciating pain*. The mean pain frequency and severity scores were calculated for each subject. Frequency scores ranged from 0 to 4, with higher scores indicating more frequent pain. Severity scores ranged from 0 to 5, with higher scores indicating greater pain severity. The values obtained for frequency and severity were standardized and then summed to yield an overall activity-related pain score.

#### Physical Function

The Short-Form Health Survey-36 (SF-36) was used to measure physical function. This form provides 8 scaled scores in the areas of physical functioning, role limitation due to physical problems, bodily pain, vitality, general health perceptions, social function, role limitations due to emotional health, and mental health (43). The subscale score for Physical functioning was used for data analysis. The SF-36 is reliable (reliability coefficients above 0.75 for all subscales except social function) and has high construct validity (44).

#### Physical Activity

The International Physical Activity Questionnaire–Long Form (IPAQ) is a subjective measure of physical activity that asks subjects to recall the amount of time doing physical activity during the past 7 days (45). Vigorous physical activity, moderate physical activity, and walking are assessed across a comprehensive set of domains including transport-related physical activity, work-related physical activity, domestic and gardening activities, and leisure-time physical activity. Guidelines provided by www.ipaq.ki.se/ipaq.htm were used for data processing and scoring of the questionnaire. Each activity was assigned a metabolic equivalent score (MET), which is based on the intensity of that activity. These MET scores were derived from the IPAQ reliability study (45) and Ainsworth et al. (46). The MET scores are then multiplied by the reported number of minutes per week spent performing that activity, which produces an activity score of METs-minute/week. Scores were calculated for vigorous activity, moderate activity, walking, and Total activity (Total PA). The test has shown acceptable concurrent and constructs validity and test-retest reliability (0.66–0.89) (45).

### Data Analysis

A power analysis using G Power (Franz Faul, Universitat Kiel, Germany) 3.0.10 was used to estimate the sample size needed for predicting the change in R^2^ in a multiple linear regression model when the independent variable of interest was added to the model. With an estimated moderate effect size (f^2^ = 0.16) and including two covariates, a sample size of 52 participants would provide the power of 0.80 at *p* = 0.05.

Descriptive statistics were calculated for age, body mass index (BMI), and all outcome measures. Shapiro-Wilk's test of normality indicated that all the data except for percent of sedentary time and percent of light physical activity were not normally distributed; thus Mann-Whitney U tests were conducted to determine if these variables differed by sex. Independent *t*-tests were conducted to determine whether the percent of sedentary time and percent of light physical activity differed by sex.

Spearman's Rho bivariate correlation analyses were conducted to determine whether TSK and activity-related pain (QWB-pain scale) were associated with age, BMI, self-reported physical function (SF-36), self-reported physical activity (IPAQ scores), PA variables derived from the accelerometers, SPPB scores, maximal handgrip, and 30-s chair stand test score. Spearman's correlations were used because most of the variables were not normally distributed. Third, hierarchical regressions were conducted to determine whether TSK predicted objective and subjective physical function and physical activity, after controlling for significant covariates (e.g., BMI, age, sex, activity-related pain). For all regressions, covariates that were significantly associated with the dependent variable (i.e., significant correlation) were entered into the model before entering the predictor of interest.

## Results

### Participants

The descriptive statistics for participant demographics and all outcome measures are presented in Table 1. The descriptive statistics are separated by sex. Target force production on the handgrip (*p* < 0.001) and percentage of wear time in LPA (*p* = 0.019) differed significantly between males and females. Males had greater force production on the handgrip and a lower percentage of wear time in LPA compared to females.

### Correlations of TSK and Activity-Related Pain With Demographics and Physical Function Variables

Age (r = −0.363, *p* = 0.008) and BMI (r = 0.320, *p* = 0.021) significantly correlated with IPAQ-Sit subscale score. BMI also significantly correlated with TSK score (r = 0.289, *p* = 0.038). Table 2 presents the correlations between TSK, activity-related pain, and the physical function-related variables. The results demonstrated significant negative associations between TSK score and 30-s Chair Stand test score, SPPB total score, and SF-36 Physical Function subscale score. Significant positive associations were found between TSK score and SPPB gait time and SPPB chair stand time. Thus, greater kinesiophobia was related to worse self-reported physical function and decreased performance on the 30-s Chair Stand test and on the gait task and repeated chair stands the task of the SPPB. The correlation analyses also showed that greater activity-related pain was significantly associated with worse performance on the balance task and the repeated chair stands task of the SPPB, a lower score for the SPPB total score, and worse self-reported physical function on the SF-36.

**Table 2:** Bivariate correlation matrix between Tampa Scale of Kinesiophophia (TSK), activity-related pain, and the physical function measures.

	**1**	**2**	**3**	**4**	**5**	**6**	**7**	**8**	**9**
1. TSK	1.00								
2. Activity related pain	0.334^*^	1.00							
3. SF-36–Physical function	−0.430^**^	−0.545^**^	1.00						
4. Handgrip MVC	−0.082	0.971	0.229	1.00					
5. SPPB–Gait speed	0.362^**^	0.246	−0.203	−0.065	1.00				
6. SPPB–Chair stand time	0.453^**^	0.342^*^	−0.351^**^	−0.087	0.408^**^	1.00			
7. SPPB–Balance score	−0.117	−0.320^*^	0.350^*^	0.112	0.041	−0.297^*^	1.00		
8. SPPB–Total score	−0.439^**^	−0.421^**^	0.472^**^	0.088	−0.236	−0.841^**^	0.469^**^	1.00	
9.30-s Chair stand test	−0.479^**^	−0.236	0.281^*^	0.159	−0.506^**^	−0.755^**^	0.099	0.588^**^	1.00

* *= p < 0.05;*

** *= p < 0.001; TSK, Tampa Scale of Kinesiophobia; MVC, Maximal voluntary contraction; SPPB, Short Physical Performance Battery*.

### Correlations of TSK and Activity-Related Pain With Physical Activity Variables

Table 3 presents the correlations between TSK, activity-related pain, and the physical activity-related variables. In regard to the accelerometer variables, greater kinesiophobia on the TSK was significantly associated with greater sedentary behavior and decreased time in light physical activity and MVPA. Activity-related pain was not significantly associated with any of the accelerometer variables. TSK scores were not significantly related to self-reported PA on the IPAQ. Greater activity-related pain was associated with decreased moderate PA on the IPAQ.

**Table 3:** Bivariate correlation matrix between TSK, activity-related pain, and the physical activity measures.

	**1**	**2**	**3**	**4**	**5**	**6**	**7**	**8**	**9**	**10**
1. TSK	1.00									
2. Activity related pain	0.334^*^	1.00								
3. IPAQ-Vigorous	−0.150	0.045	1.00							
4. IPAQ-Moderate	−0.196	−0.309^*^	0.071	1.00						
5. IPAQ-Walking	−0.109	−0.214	0.399^**^	0.443^**^	1.00					
6. IPAQ-Sit	0.085	0.254	−0.091	−0.395^**^	−0.244	1.00				
7. IPAQ-Total	−0.221	−0.249	0.509^**^	0.797^**^	0.769^**^	−0.305^*^	1.00			
8. % of Sedentary time	0.458^**^	0.215	0.033	−0.175	−0.136	0.109	−0.162	1.00		
9. % of LPA	−0.415^**^	−0.160	−0.122	0.126	0.014	−0.095	0.060	−0.951^**^	1.00	
10. Minutes in MVPA	−0.319^*^	−0.258	0.426^**^	0.152	0.389^**^	−0.092	0.357^**^	−0.347^*^	0.074	1.00

* *= p < 0.05;*

** *= p < 0.001; TSK, Tampa Scale of Kinesiophobia; IPAQ, International Physical Activity Questionnaire; LPA, Light physical activity; MVPA, moderate to vigorous physical activity*.

### Hierarchical Regressions

#### Self-Reported Physical Function

After controlling for BMI, age, and activity-related pain, TSK predicted self-reported physical function on the SF-36 (Table 4). Older adults who reported greater kinesiophobia reported worse physical function. In this model, activity-related pain accounted for the highest proportion of the variance at 38%.

**Table 4:** Summary of hierarchical regression analyses for self-reported and performance-based physical function variables with TSK as final predictor.

**Step variables**	**ΔR^**2**^**	**Standardized β**	***P*** **-value for β**	**Model *p*-Value**
**A. SF-36 physical function subscale**				
1. Age	0.036	0.053	0.617	<0.001
BMI		−0.004	0.970	
2. Activity-related pain	0.382	−0.529	<0.001	
3. TSK	0.070	−0.291	0.015	
**B. 30-s Chair stand test score**				
1. TSK	0.200	−0.447	0.001	0.001
**C. SPPB–gait speed**
1. Activity related pain	0.149	0.206	0.102	<0.001
2. TSK	0.204	0.486	<0.001	
**D. SPPB–chair stand time**				
1. Activity related pain	0.095	0.153	0.257	<0.001
2. TSK	0.152	0.420	0.003	
**E. SPPB–total score**
1. Activity related pain	0.151	−0.237	0.072	<0.001
2. TSK	0.145	−0.410	0.003	

*TSK, Tampa Scale of Kinesiophobia*.

#### Self-Reported Physical Activity

TSK was not associated with any of the IPAQ variables in the correlation analysis, and thus was not further investigated as a potential predictor of self-reported PA. The only IPAQ variable that correlated with any of our predictor variables was the Moderate PA subscale; therefore, this was the only IPAQ variable on which we conducted a regression. In the regression analysis (model *p* = 0.038), activity-related pain significantly predicted moderate PA accounting for 8.3% of the variance (Beta = −0.289, *p* = 0.038).

#### Performance-Based Measures of Physical Function

Performance on the maximal handgrip test was not significantly correlated with any variables; thus, a regression to predict handgrip performance was not conducted. The regression model for the 30-s Chair Stand test was significant, with greater TSK predicting fewer chair stands on the 30-s Chair Stand test (Table 4). The regression model for the Balance score on the SPPB with activity-related pain as the predictor was not significant, *p* = 0.051 (TSK was not included as a predictor because it was not significantly correlated with the Balance score). As the SPPB Gait score and Gait time represent the same task, we only conducted regression analysis on the SPPB Gait time. After controlling for activity-related pain, TSK significantly predicted SPPB Gait speed (Table 4). Greater activity-related pain and TSK were associated with slower gait speed times. The entire model accounted for 33% of the variance. In a separate regression, activity-related pain and TSK also predicted SPPB Chair Stand time (Table 4). Older adults reporting greater TSK and activity-related pain took longer to complete the 5 chair stands. Additionally, after controlling for activity-related pain, TSK significantly predicted the Total SPPB score (Table 4). Greater TSK was associated with a lower total score on the SPPB.

#### Objective Measures of Physical Activity

The objective measures of PA included the three variables (Percentage of time in sedentary time and light physical activity, and MVPA) derived from the accelerometers. The regression model for each variable was significant. After controlling for BMI, the regression indicated that TSK significantly predicted the percentage of wear time spent being sedentary (Table 5), accounting for 16.7% of the variance. Older adults who reported greater kinesiophobia exhibited greater sedentary behavior. After controlling for sex, TSK also predicted the percentage of time spent in light physical activity with greater kinesiophobia associated with lower light physical activity (Table 5). Finally, after controlling for BMI, TSK significantly predicted MVPA, accounting for 6.9% of the variance (Table 5). Older adults who reported greater kinesiophobia did less MVPA.

**Table 5:** Summary of hierarchical regression analyses for physical activity variables with TSK as final predictor.

**Step variables**	**ΔR^**2**^**	**Standardized β**	***P*** **-value for β**	**Model *p*-Value**
**A. Percentage of sedentary time**				
1. BMI	0.066	0.156	0.230	0.002
2. TSK	0.167	0.420	0.002	
**B. Percentage of light physical activity**				
1. Sex	0.120	0.297	0.022	0.001
2. TSK	0.120	−0.350	0.008	
**C. MVPA**
1. BMI	0.140	−0.310	0.022	0.003
2. TSK	0.069	−0.271	0.044	

*TSK, Tampa Scale of Kinesiophobia; MVPA, moderate to vigorous physical activity*.

## Discussion

The impact of fear of movement on physical function and activity in healthy older adult populations has received little attention. The current study provided novel evidence of the relationship between kinesiophobia and different facets of physical function in healthy, chronic pain-free older adults, including self-reported physical function, performance-based function, and objective levels of physical activity and sedentary behavior. Several key findings emerged from the results. First, in regard to patient-reported outcomes, kinesiophobia predicted physical function but not physical activity levels. Second, kinesiophobia predicted outcomes on performance-based tests of lower-extremity physical function, even after controlling for activity-related pain. Third, kinesiophobia also predicted objective measures of physical activity, including sedentary time, light physical activity, and MVPA. Generally, older adults with decreased pain-related fear of movement reported better physical function, exhibited increased lower extremity physical function, and were more physically active. This study also highlights the importance of comprehensive physical function and physical activity assessment when investigating kinesiophobia, pain, and physical function.

As hypothesized, the results from our study revealed that kinesiophobia predicted physical function in healthy, chronic pain-free older adults, as indicated by patient-reported outcomes and performance-based measures of functional capacity. After controlling for activity-related pain, older adults with greater pain-related fear of movement reported increased limitations on mobility activities on the SF-36. This result is in line with several studies showing kinesiophobia is associated with greater self-reported disability in patients with low back pain (47, 48), heterogeneous chronic pain (49), and knee osteoarthritis (14). Thus, based on the collective evidence, kinesiophobia appears to negatively affect middle-aged and older adults' perceptions of their own functional wellbeing, regardless of pain status. Future research needs to explore the mechanisms through which fear of movement may develop in older adults who do not have chronic pain. Perhaps, as people age, the fear of injury with movement naturally increases or even low levels of pain lead to catastrophizing and the beginning of alterations in physical behavior to avoid future exacerbation of pain. However, this is speculation and needs further study.

Our study also showed that greater fear of movement predicted poorer performance on performance-based measures of physical function as evidenced by fewer chair-stands in 30 s, slower chair-stand speed, slower gait speed, and overall lower total scores on the SPPB. However, kinesiophobia did not correlate with the measure of handgrip strength or balance on the SPPB. Thus, kinesiophobia was related to measures involving lower extremity strength, but not balance or upper extremity strength. As our results also show kinesiophobia was also related to ambulatory activity *via* the accelerometers, it is possible that fear of movement has a stronger relationship with lower extremity compared to upper-extremity activities due to the increased severity of negative outcomes (e.g., falling). These results are similar to a study conducted by Tkachuck and colleagues who showed that kinesiophobia uniquely predicted sit-to-stand and stair climbing performance in a heterogeneous sample of patients with chronic pain (35). The data on the effect of kinesophobia on other performance-based measures has been mixed. Studies have shown that kinesiophobia is related to the functional capacity of the trunk flexors and extensors in low back pain (47), but not related to functional capacity on the 6-min Walk Test in individuals with Parkinson's Disease (50) or heterogeneous chronic pain (49) or objective function in women with patellofemoral pain (51).

The physical activity data of the current study partially supported our hypothesis that kinesiophobia would predict physical activity levels in older adults. Scores on the TSK predicted the objective accelerometer measures of physical activity but not participants' perceptions of their physical activity levels. Accelerometers worn on the hip provide objective estimates of primarily ambulatory activity at various intensities but may miss activities primarily using the upper body. In regards to the accelerometer measures, greater fear of movement predicted greater sedentary time, less light physical activity, and less MVPA in our older adult sample. Interestingly, kinesiophobia had the strongest relationship with sedentary time followed by light physical activity. Few studies have examined kinesiophobia in relation to objective measures of sedentary time. Understanding factors that contribute to sedentary behavior in older adults is important because sedentary behavior has emerged as a new risk factor for many different health conditions (i.e., cardiovascular disease, diabetes), mortality, declining physical functioning, and greater disability in activities of daily living, independent of time spent in MVPA (52–54).

In contrast to the current study, prior studies have found no relationship between accelerometer-derived measures of physical activity and kinesiophobia in patients with chronic non-specific low back pain (48) and chronic musculoskeletal pain (55). Several factors could account for the contrasting results between the current study and aforementioned research. While only speculation, perhaps actual pain rather than pain-related fear of movement is a more significant inhibitor of ambulatory physical activity in individuals with chronic pain. Future research should investigate the relationship between physical activity, kinesiophobia, and activity-related pain in older adults with and without chronic pain in the same study, so that a direct comparison of these relationships can be evaluated in older adult chronic pain and chronic pain-free samples. Additionally, the current study sample included older adults, while the other studies included younger and middle-aged adults. Also, neither study of the chronic pain patients measured sedentary behavior and one study only measured the mean activity count. The age of participants, as well as differences in accelerometer-derived measures of physical activity, could influence the degree of relationship between kinesiophobia and objective measures of physical activity.

Self-reported measures of physical activity reflect the participants' perception of their activity levels and can capture activities of daily living that are not captured with accelerometers. Generally, the evidence is mixed regarding the association of kinesiophobia with patient-reported outcomes of physical activity. In line with the current study, prior research has demonstrated no relationship between self-reported measures of physical activity and kinesiophobia in primary health care patients with musculoskeletal pain (56), younger adults with temporomandibular disorder (57), and patients with chronic neck pain (58). However, significant research has also found that greater kinesiophobia is associated with lower levels of self-reported physical activity in individuals with knee osteoarthritis (14, 15), non-specified chronic pain (59), chronic low back pain (60), Parkinson's Disease (61), and a general adult population (18). It remains unclear why such discrepant results exist regarding the relationship between self-reported physical activity and kinesiophobia, but the wide variety of self-reported measures likely plays a role. For example, the questionnaires vary in the dimensions of physical activity assessed (frequency, intensity, etc.), the type of physical activity assessed (e.g., leisure time vs. exercise), and in complexity ranging from validated questionnaires to a few author-developed questions.

Several limitations of this study should be acknowledged. First, due to the cross-sectional nature of the study, we cannot assume that kinesiophobia causes worse physical function and sedentary behavior in older adults. Future longitudinal research is needed to verify the causal relationship between kinesiophobia and the different facets of physical function in older adults. Secondly, the sample of participants in the current study consisted of healthy older adults, who were primarily Caucasian and likely more active than the average older adult. Therefore, generalization of the results to more sedentary older adults and those of other ethnic backgrounds may be limited. Additionally, the study sample did not include older adults above the age of 80 years. It is possible that the relationship between kinesiophobia and sedentary behavior could be further magnified in a more sedentary or older sample. Third, some of the variance percentages were low for TSK in the significant regression models, particularly for self-reported physical function and MVPA measured *via* accelerometry. This indicates that there are other significant factors that predict physical activity and function in this population that were not measured in the current study. It should also be noted that the TSK scores of the current sample were relatively low (range 11–31, with the average score around 17–18), as would be expected of a sample of adults without reported chronic pain. Comparatively, in the validation study of the TSK-11, average scores were in the low 30's (max score is 44) in a sample of middle-aged adults with heterogeneous chronic pain (36). In a large study of older adults with heterogeneous chronic pain, average TSK-11 scores were in the low 20's (59). Thus, our study shows that even lower levels of fear of movement can impact physical function in healthy, chronic pain-free older adults. Fourth, physical activity behavior was only assessed over a seven-day period, which may not have been representative of overall physical activity habits for each participant. Finally, activity-related pain was measured with a self-reported questionnaire that was based on recall. Future studies should include momentary pain assessments during on-site physical function tests to collect a more accurate assessment of activity-related pain (also referred to as movement-evoked pain).

In conclusion, fear of movement due to pain or reinjury is maladaptively associated with decreased functional wellbeing, functional capacity and physical activity levels, even in older adults seemingly healthy and chronic pain free. These results support the hypothesis that fear of movement may lead to reduced functional capacity and inactivity in older adults. Accordingly, perhaps fear of movement should be assessed by clinicians in all older adults, regardless of pain status, as this may be a risk factor for a slow decline in physical activity and function. Additionally, future research should investigate the mechanisms leading to fear of movement in older adults without chronic pain, so that clinicians can work to prevent this debilitating mindset.

## Data Availability Statement

The raw data supporting the conclusions of this article will be made available by the authors, without undue reservation.

## Ethics Statement

The studies involving human participants were reviewed and approved by Indiana University Institutional Review Board. The patients/participants provided their written informed consent to participate in this study.

## Author Contributions

KMN, KEN, ZR, and NK contributed to the study design. KMN supervised data collection and performed data analysis. KMN and CB drafted the manuscript and performed data interpretation. KEN, ZR, and NK provided critical revisions. All authors approved the final version of the manuscript for submission.

## Funding

This research was supported by the IUPUI School of Health and Human Sciences Faculty Research Opportunity Grant.

## Conflict of Interest

The authors declare that the research was conducted in the absence of any commercial or financial relationships that could be construed as a potential conflict of interest.

## Publisher's Note

All claims expressed in this article are solely those of the authors and do not necessarily represent those of their affiliated organizations, or those of the publisher, the editors and the reviewers. Any product that may be evaluated in this article, or claim that may be made by its manufacturer, is not guaranteed or endorsed by the publisher.

## Abbreviations

TSK: Tampa Scale of Kinesiophobia
QST: quantitative sensory testing
SPPB: Short Physical Performance Battery
MVC: Maximum voluntary contraction
MVPA: Moderate to vigorous physical activity
LPA: Light physical activity
QWB-SA: Quality of Wellbeing Scale – Self Administered
SF-36: Short Form health Survey-36
IPAQ: International Physical Activity Questionnaire
BMI: Body Mass Index
PA: Physical activity.
